# Integrating a framework for conducting public health systems research into statewide operations-based exercises to improve emergency preparedness

**DOI:** 10.1186/1471-2458-12-680

**Published:** 2012-08-20

**Authors:** Jennifer C Hunter, Jane E Yang, Michael Petrie, Tomás J Aragón

**Affiliations:** 1 School of Public Health, University of California, Berkeley, CA, USA; 2 County of Santa Clara Emergency Medical Services Agency, San Jose, CA, USA; 3 San Francisco Department of Public Health, San Francisco, CA, USA

**Keywords:** Activities, Capabilities, Emergency response, Exercises, Functions, Information sharing, Inter-organizational communications, Preparedness, Systems research

## Abstract

**Background:**

Due to the uncommon nature of large-scale disasters and emergencies, public health practitioners often turn to simulated emergencies, known as “exercises”, for preparedness assessment and improvement. Under the right conditions, exercises can also be used to conduct original public health systems research. This paper describes the integration of a research framework into a statewide operations-based exercise program in California as a systems-based approach for studying public health emergency preparedness and response.

**Methods:**

We developed a research framework based on the premise that operations-based exercises conducted by medical and public health agencies can be described using epidemiologic concepts. Using this framework, we conducted a survey of key local and regional medical and health agencies throughout California following the 2010 Statewide Medical and Health Exercise. The survey evaluated: (1) the emergency preparedness capabilities activated and functions performed in response to the emergency scenario, and (2) the major challenges to inter-organizational communications and information management.

**Results:**

Thirty-five local health departments (LHDs), 24 local emergency medical services (EMS) agencies, 121 hospitals, and 5 Regional Disaster Medical and Health Coordinators/Specialists (RDMHC) responded to our survey, representing 57%, 77%, 26% and 83%, respectively, of target agencies in California. We found two sets of response capabilities were activated during the 2010 Statewide Exercise: a set of core capabilities that were common across all agencies, and a set of agency-specific capabilities that were more common among certain agency types. With respect to one response capability in particular, inter-organizational information sharing, we found that the majority of respondents’ comments were related to the complete or partial failure of communications equipment or systems.

**Conclusions:**

Using the 2010 Statewide Exercise in California as an opportunity to develop our research framework, we characterized several aspects of the public health and medical system’s response to a standardized emergency scenario. From a research perspective, this study provides a potential new framework for conducting exercise-based research. From a practitioner’s perspective, our results provide a starting point for preparedness professionals’ dialogue about expected and actual organizational roles, responsibilities, and resource capacities within the public health system. Additionally, the identification of specific challenges to inter-organizational communications and information management offer specific areas for intervention.

## Background

The infrequent nature of large-scale public health emergencies is often cited as a barrier to preparedness evaluation and improvement. With few events, researchers have limited use of statistical methods to test hypotheses and to identify predictors of effective response outcomes—the characteristics of public health systems that result in the fewest number of adverse health outcomes at the least cost to society [1]. Public health practitioners increasingly rely on simulated emergencies, known as “exercises”, for preparedness assessment and improvement. Exercises routinely play an important role in building and testing emergency response capabilities [2]. Under the right conditions, they can also be used to conduct original public health systems research, functioning as “epidemiologic laboratories” where participants are exposed to scenarios and injects that can answer high-priority, all-hazards preparedness and response research questions.

Although exercises have frequently served as a platform for studying public health emergency preparedness (e.g. [3-5]), the potential for exercise-based research to produce generalizable evidence in emergency preparedness has not been fully realized. There are several possible reasons for the under-utilization of this resource. First, there are currently no well-defined, universal standards for public health emergency preparedness, limiting our ability to compare agency or system performance with a recognized benchmark or metric [6]. As a result, findings from exercise-based research have generally been limited to summaries of strengths and limitations experienced by participants. Second, to realistically test preparedness systems, it is necessary to conduct operations-based exercises, during which participants actually respond to the simulated emergency [7].^a^ However, due to the resource-intensive nature of developing and conducting this type of exercise, researchers either: (1) rely on more common and less costly discussion-based exercises, which are useful for identifying emergency preparedness gaps and vulnerabilities but do not directly test the preparedness system [8], (2) evaluate exercises with a limited number of participating jurisdictions, restricting the generalizability of research findings and lessons for systems improvement [3], or (3) aggregate data from multiple exercises with various scenarios in order to assess common themes and challenges [4,5].

In this paper, we describe the integration of a research framework to a statewide functional exercise in California. By leveraging the pre-existing statewide exercise program, we were able to access a relatively large number of medical and health agencies at a low cost, allowing us to gain a broader view of how public health systems in California operate in response to a single scenario.

### Statewide exercise

The Medical and Health Exercise (hereafter referred to as the “Statewide Exercise”) is one of two annual emergency preparedness exercises coordinated at the state level in California. During this multi-level, multi-agency exercise, all 58 operational areas^b^ and 6 regional offices in California are invited to participate in functional exercises in collaboration with state agencies using a single common emergency scenario [9]. Each operational area designs and conducts the exercise to suit its own preparedness goals and objectives, either conducting the exercise independently or in collaboration with other jurisdictions. Although exercise participation varies among operational areas, certain agencies such as hospitals, public health departments and emergency medical services (EMS) agencies are incentivised to participate to meet grant or regulatory requirements.

The 2010 exercise scenario involved a series of explosions throughout the state resulting from the detonation of improvised explosive devices (IED).^c^ While each operational area was expected to tailor their exercise to meet their own needs, all participants were encouraged to use the exercise to activate capabilities related to communications, information sharing, and medical surge in response to bomb threats, explosions, and multiple casualties. A common exercise guidebook was developed by state agencies to aid in the coordination of scenario development [10]. Using this as an opportunity to develop our research framework, we distributed a post-exercise survey and conducted a mixed-methods study of two research questions:

1. Which emergency preparedness capabilities were activated by entities in the medical and health system in response to the exercise scenario?

2. What were the major challenges to inter-organizational communications and information sharing?

The primary aim of this paper is to describe the process of conducting research using statewide exercises. By presenting the results of two example research questions, we illustrate the type of data that can be collected using this approach, and highlight the opportunities and challenges for future research.

## Methods

### An epidemiologic exercise model

**Figure 1:**
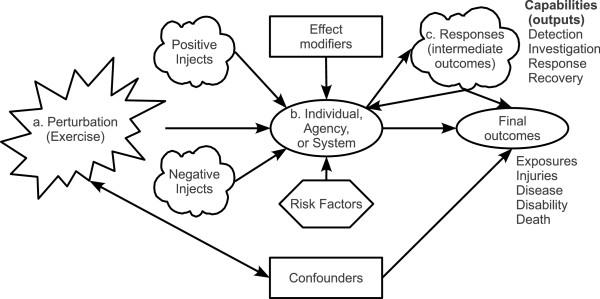
**Epidemiologic Exercise Model. **Figure 1 shows an operations-based exercise that can form the basis of a variety of study designs, such as a retrospective or prospective observational study. Sections labelled with a, b, and c indicate the simplified version of the model we used as a first step in actualizing the research framework. Notice that both the exercise scenario (perturbation) and/or injects can be randomly allocated to participants in order to conduct a randomized controlled trial.

Our research framework is based on the premise that operations-based exercises can be described using epidemiologic concepts (Figure 1). As a first step in actualizing the research framework, we used a simplified version of this model, represented by the letters a, b and c. The exercise scenario (denoted by a) constitutes the system perturbation or exposure, which can vary with different exercise injects. Upon receipt of the perturbation and injects, the exercise participants or agencies (denoted by b) perform actions (responses or intermediate outcomes, denoted by c) that directly or indirectly result in final outcomes, such as injury, disease, disability, and death.

Using the epidemiologic exercise model (Figure 1) as a guide, various types of study designs (randomized controlled trial, cross-sectional, etc.) can be implemented in the right exercise setting. The research possibilities are enormous and have not yet been fully tapped. To provide an example, we describe our method of studying two research priorities identified by our practice-based Steering Committee, which is composed of decision-makers in state and local public health and medical agencies. The intent of engaging a practice-based Steering Committee was to develop research priorities and questions relevant to practice, and to facilitate research translation into policy or practice [11].

### Study design and instrument

Using a cross-sectional study design, we conducted a web-based survey to evaluate four domains related to the Statewide Exercise. The two described in this paper are: (1) organizational capabilities and functions and (2) challenges to inter-organizational communications and information sharing. In the context of our epidemiologic exercise model, these domains represent two ways of characterizing of response activities, which are conceptualized as intermediate outcomes in the causal pathway from the exercise perturbation event to final outcomes (Figure 1).

Throughout its development, the survey instrument was periodically reviewed by the Steering Committee and pilot-tested by representatives from participating organizations. Subsequent modifications were made based on this feedback.

### Measurements

#### Capabilities and functions

As a proxy measure of organizational roles and responsibilities assumed during a response—areas previously identified as requiring further research and improvement [1]—we assessed the capabilities and functions activated by public health and medical agencies during the Statewide Exercise. Standard response capabilities and functions were introduced by the Department of Homeland Security Target Capabilities List, which defines activities and tasks that form the basis of performance metrics and benchmark criteria to assess preparedness levels [12]. The term capability refers to the ability to perform functions or activities necessary for an effective response to major disasters and emergencies. Survey respondents were asked to indicate whether persons in their organization were assigned to functions related to each of 33 capabilities. Common capabilities were excluded from the survey, since they are cross-cutting and expected to be engaged in every response. Psychological support was added based on researchers’ interests.

#### Inter-organizational communications & information sharing challenges

To identify challenges to inter-organizational communications and information sharing, the survey included two questions. The first was a subjective and open-ended question, asking respondents to describe their most significant communications challenge during the exercise. The second instructed respondents to select the types of communication challenges their organization experienced from a list of options that was developed from the literature and Steering Committee members’ experiences.

#### Exercise participation

Survey respondents were also asked about the types of exercises conducted during the 2010 Statewide Exercise, characteristics of participating agencies, and factors influencing exercise participation. This information has been published elsewhere [13].

### Study population

To evaluate California’s medical and public health system during the Statewide Exercise, representatives from all local health departments (LHDs; n = 61), local EMS agencies (n = 31), and Regional Disaster Medical and Health Coordinator/Specialists (RDMHC; n = 6) were invited to participate in the post-exercise survey. Since contact information for hospital preparedness staff is not publicly available, we relied on the assistance of the California Hospital Association (CHA) to recruit all general acute care hospitals (n = 466) in California, which comprise 87% of all licensed hospitals in the state (n = 534).

During a one-month data collection period following the Statewide Exercise, survey invitations and three subsequent reminders were emailed using a web-based system (Qualtrics^©^). Survey recipients were chosen based on their functional role in their organization: health officers for LHDs, administrators for local EMS agencies, and preparedness coordinators for hospitals. Whereas government agency representatives directly received invitations and reminders from the Principal Investigator, hospitals received such communications as a forwarded message from CHA.

### Data management and analyses

Survey data were restricted by date range and analyzed using Stata 11 (StataCorp LP, College Station, TX). The response rate was calculated after limiting the dataset to respondents who partially or fully completed the survey, and removing duplicate responses—the result of having multiple respondents from the same organization or from the same region, in the case of respondents who were RDMHC. When duplicate responses were found, researchers used a pre-determined prioritization scheme based on survey completion status and target functional role for an agency type (e.g., health officers were the intended functional role for LHDs) to determine which response to include.

#### Agency classification

We used survey respondents’ self-designation to classify agency types. Five types of respondents emerged—those who declared an affiliation with: (1) LHDs, (2) local EMS agencies, (3) LHDs and local EMS agencies, (4) hospitals, and (5) RDMHC. Follow-up interviews were conducted with respondents who identified an affiliation with both LHDs and EMS agencies, all of whom affirmed their responses reflected both agencies’ experiences during the exercise. Since these respondents were affiliated with agencies legally recognized as local EMS agencies that operate within a LHD (California Health and Safety Code Section 1797.200), this category is hereafter referred to as “local EMS agency within a LHD.”^d^

#### Quantitative analysis

For both research questions, analysis was restricted to data from respondents who met the following criteria: (1) partially or fully completed the survey, (2) indicated their agency participated in the Statewide Exercise using the common scenario, and (3) indicated their agency carried out an operations-based exercise.

**Figure 2:**
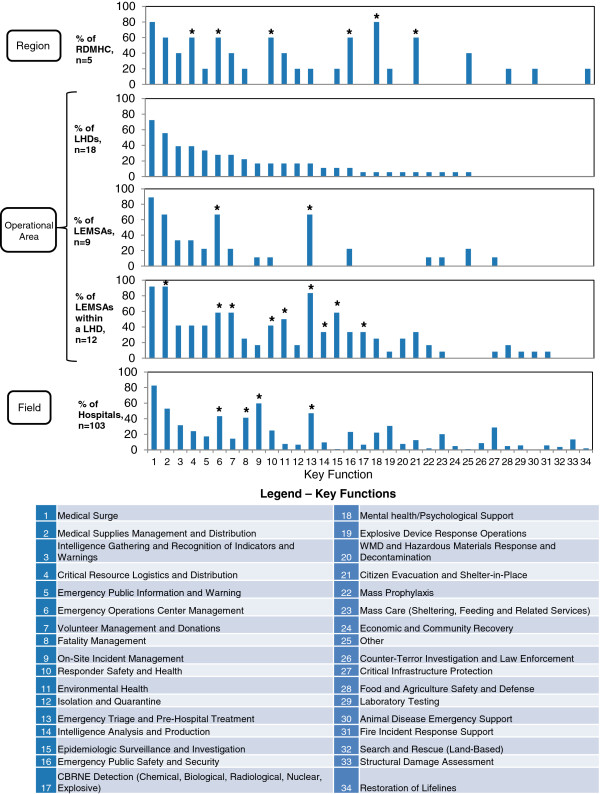
**Agency-specific Profiles of Activated Key Capabilities. **Figure 2 shows a graphical profile of public health capabilities activated by different agency types during the Statewide Exercise. For each of the graphs, the x-axis is numbered from 1 to 34, which are the numerical codes for the key capabilities defined in the Department of Homeland Security’s Target Capabilities List, 2007 (see legend). The y-axis for each graph indicates the percentage of the agency type that activated a given key capability. LHDs were treated as the “baseline”; its activated key capabilities were sorted in descending order of frequency, and the other agency types were arranged accordingly. The asterisk (*) indicates key capabilities reported by agencies other than LHDs that notably differed from LHDs. (Note: LEMSAs = local EMS agencies).

In order to characterize the capabilities and functions activated in response to the exercise scenario, we used the frequency of reported activation to find the average number and range of capabilities for each agency type. Capabilities that were commonly activated for all agency types, as well as those characteristic of a specific agency type, were identified via graphical response profiles (Figure 2).

#### Qualitative analysis

Qualitative data were independently coded by two researchers (JH, MP) to classify statements into themes, categories, and sub-categories. All coding discrepancies were resolved. Descriptive summaries of themes are provided and supplemented by illustrative quotes (see Additional file 1). These analyses were further informed by direct observation of the exercise by researchers.

This research was approved by the Committee for Protection of Human Subjects at the University of California, Berkeley.

## Results

In the following section, we summarize the results of two example questions in effort to demonstrate the type of data that can be collected using this research framework and to underscore potential challenges and opportunities that may arise using this approach. Detailed results for both research questions can be found in Additional file 1.

### Respondent and organizational demographics

Our study sample includes 174 respondents who represent 35 LHDs, 24 local EMS agencies, 121 hospitals, and 5 RDMHC in the state, giving response rates of 57%, 77%, 26% and 83%, respectively ^e,f^. Of 174 respondents, 144 met the inclusion criteria for analysis of the questions relevant to this paper. These represent 30 LHDs, 21 local EMS agencies, 103 hospitals, and 5 RDMHC, which constitute 49%, 68%, 19% and 83%, respectively, of the target populations.^g^

The final sample includes responses from agencies located in 46 of the 58 counties in California (79%). All six Mutual Aid Regions were represented by responding LHDs, local EMS agencies, and RDMHC (at least 35% of agencies in each Region responded). The most common functional roles of respondents were health officers or health directors (39% of LHDs), preparedness coordinators (50% of LHDs, 33% of local EMS agencies, 42% of local EMS agencies within a LHD, and 90% of hospitals), and administrators (44% of local EMS agencies and 92% of local EMS agencies within a LHD).

To assess the representativeness of our research sample, we compared key demographic characteristics of counties from which we received responses to counties that did not respond (Table 1). T-tests and chi-square tests showed that LHD response rates did not vary (at the p < 0.05 level) in terms of population size, county sizes, and level of urbanity. Chi-square tests indicated that local EMS agency response rates differed (at the p < 0.05 level) by county size; medium-sized counties in California are under-represented in the survey results. T-tests showed that hospital response rates varied (at the p < 0.05 level) by the median number of licensed hospital beds, which suggests smaller hospitals may be under-represented by survey results.

**Table 1:** Demographic Characteristics of Responding versus Non-Responding Agencies*

	**Responding**			**Non-Responding**		
	LHDs (n=32)**	Local EMS Agencies (n=42)***	Hospitals (n=121)****	LHDs (n=29)**	Local EMS Agencies (n=16)***	Hospitals (n=391)****
**Median Population Size Served** †	228,618	183,427	---	126,518	127,645	---
**Median # Licensed Hospital Beds ††**	---	---	166	---	---	114
**County Size** §						
Small	25%	31%	---	24%	13%	---
Medium	47%	38%	---	52%	75%	---
Large	28%	31%	---	24%	13%	---
**Mean Level of Urbanity** ¥	1.5	1.52	---	1.41	1.38	---

* Since hospitals do not serve an entire county, we compare the median number of licensed hospital beds between responding and non-responding hospitals.

** LHDs representing 32 of 61 health jurisdictions responded to the post-exercise survey; 29 health jurisdictions are not represented.

*** 24 LEMSAS representing 42 counties responded to the post-exercise survey; 16 counties are not represented.

**** 466 general acute care hospitals in California were recruited for the survey, 121 of which responded. An additional 6 hospitals (3 federal hospitals and 3 acute psychiatric hospitals) were included in the analysis, for a total of 127 hospitals.

† Based on data from the U.S. Census Bureau, 2000.

‡ Based on data from the Office of Statewide Health Planning and Development, part of the California Health and Human Services Agency.

§ Small LHD = population < 50,000; Medium LHD = population 50,000-499,999; Large LHD = population ≥500,000.

¥ 1 = Primarily Urban, 2 = Urban/Rural Mix, 3 = Primarily Rural.

### Key capabilities and functions

In this research, we characterize the response to the simulated IED emergency by the number and type of response capabilities (conceptualized as intermediate outcomes in our epidemiologic exercise model; Figure 1, letter c) activated by each agency and the role of each agency (i.e., lead versus supporting). Figure 2 provides a profile of response capabilities activated during the Statewide Exercise. Two sets of capabilities are depicted: first, a set of core capabilities activated across all agencies in response to the exercise scenario; second, a set of agency-specific capabilities, which were more common among certain types of agencies. The average number of capabilities reported was highest for local EMS agencies within a LHD (mean: 9.8, range: 3–18), followed by RDMHC (mean: 8.6, range: 1–17), hospitals (mean: 6.7, range: 1–24), local EMS agencies (mean: 5.0, range: 2–7), and LHDs (mean: 4.8, range: 1–10).

As Figure 2 shows, the core capabilities commonly activated across all agencies consist of *medical surge*, *medical supplies management and distribution*, *critical resource logistics and distribution*, *emergency operations center management*, and *intelligence gathering and recognition of indicators and warnings*. In terms of agency-specific capabilities, both types of local EMS agencies and hospitals commonly activated *emergency triage and pre-hospital treatment*. LHDs and local EMS agencies within a LHD commonly activated *emergency public information and warning*. Lastly, RDMHC and local EMS agencies within a LHD commonly activated *responder safety and health*, *environmental health*, *emergency public safety and security*, and *citizen evacuation and shelter-in-place*.

For more detailed results on key capabilities commonly activated across agency types, see Additional file 1. Additional file 1: Table S2 in the appendix summarizes the proportion of each agency type that activated each function during the exercise.

More subjectively, we describe the response by asking whether agencies played a lead or supporting role during the Statewide Exercise. Two-thirds (67%) of local EMS agencies and local EMS agencies within a LHD stated they played a lead role, while the majority of LHDs, hospitals, and RDMHC indicated they played a supporting role (61%, 76%, and 60%, respectively).

#### Challenges to inter-organizational communications and information sharing

Looking specifically at one response capability, inter-organizational information sharing, we characterize specific challenges, which if removed, would be expected to improve final outcomes. The majority (56%) of respondents’ comments referred to challenges related to the complete or partial failure of communications equipment or systems—specifically an internet-based hospital status system which did not operate as expected, making it necessary for staff to collect data manually, repeatedly enter information, or rely on a back-up system (e.g., radio, fax, or runner) in order to receive or input information. Results on the most significant communications challenge encountered during the exercise, as well as challenges to inter-organizational communications and information sharing, are detailed in Additional file 2: Figure S3.

## Discussion

This paper describes the application of a conceptual framework to a Statewide Exercise as a systems-based approach for studying public health emergency preparedness and response. Capitalizing on a pre-existing statewide operations-based exercise program, we were able to access a large population of medical and health agencies with minimal cost, and to characterize several aspects of the public health and medical response to the standardized emergency scenario. Our research findings may provide an evidence-based starting point for preparedness professionals’ dialogue about expected and actual organizational roles, responsibilities, and resource capacities within the public health system—an area that needs improvement, according the literature and our Steering Committee [1]. Additionally, we identify specific challenges to inter-organizational communications and information sharing, which could provide specific areas for intervention.

To our knowledge, this represents the first attempt to develop and integrate a comprehensive research framework into the conduct of a statewide operations-based exercise. Attributes of this research framework that lend it unique strengths include: (1) having sufficient organizational units of analysis to test statistical hypotheses, (2) the ability to apply epidemiologic concepts to identify which public health system characteristics are associated with positive health outcomes, and (3) the ability to test hypotheses using randomized controlled trials in which agencies are randomly allocated different scenarios or injects during exercise play. Because this is a novel approach to conducting exercise-based preparedness research, in the following section, we describe the lessons learned and challenges faced.

### Challenges and opportunities

We were only able to partially operationalize our research framework for this Statewide Exercise, describing the perturbation (exercise) and intermediate outcomes (response activities) for different agencies within the public health system. To fully leverage the Statewide Exercise for the purpose of answering preparedness questions, a randomized controlled trial should be conducted by randomly allocating injects to agencies during exercise play. For example, positive or negative injects can be randomly assigned to agencies to measure exposure-response effects. Conducting such a study would require organizational buy-in from the exercise planning group and exercise participants at an early stage of exercise design and would require researchers to be particularly attentive to factors that might confound the exposure-response effects, including the local implementation of the exercise scenario, characteristics of the community served, and the distribution of public health responsibilities within a community. Under the right conditions, the approach we have described can be used to demonstrate variations in performance outcomes, such as the speed, quality, and the equitable provision of public health emergency services, and ultimately, to identify factors associated with high performing health departments.

In our study, we narrowly defined California’s public health and medical preparedness system to include local public health and EMS agencies, regional medical and health disaster coordinators, and hospitals. Despite this conservative definition, the system is complex, and includes the 61 LHDs, 31 local EMS agencies, 58 operational areas, 6 disaster medical and health regions, and 534 hospitals. In many organizations, emergency preparedness functions are not a full-time or a primary job responsibility, and individuals may fill the preparedness role for multiple organizations within the same county (e.g., EMS and public health agencies) or for multiple counties. Additionally, operations centers are often multi-use facilities, serving Department Operations Centers and Regional Operations Centers (e.g., the RDMHC may fill the regional disaster medical health role in a facility shared with an EMS agency). The overlapping roles of preparedness professionals in this system presented notable challenges in recruitment and data analysis, underscoring the complexity of California’s medical and health system and the subsequent challenge of assessing differences across agency types, which are not always clearly differentiated. Until the system components are better understood, the use of a web-based survey may not adequately capture how response functions are organized and delivered within the public health and medical system.

Although the integration of a research framework within the Statewide Exercise program yielded advantages of a larger sample size at a relatively low cost, there are several limitations. First, although state agencies recommend that operational areas include certain common features in their exercise design, each jurisdiction has the autonomy to develop an exercise that best meets their needs. Consequently, there is wide variation in the characteristics of exercises conducted across the state. To control for this, we limited our analysis to respondents who performed an operations-based exercise using the designated exercise scenario, on the specified day. However, additional variation in the exercise design (including the size, scope, and location of the emergency scenario, and the level of exercise participation) could not be controlled for. As a result, it is not clear how much of the variability in the observed response is attributable to differences in local implementation. To improve the strength and validity of findings, we would recommend additional standardization of future exercise characteristics at the local level, as well as incorporating random allocation of exercise injects to agencies sharing similar characteristics in terms of organizational structure, community served, and public health responsibilities. However, we acknowledge that this might be a challenge, particularly because exercises of this scale involve many agencies, and health departments may not have control over important aspects of exercise planning and implementation.

Second, because all operational areas were encouraged to simultaneously conduct exercises on the same day, the number of IED attacks “experienced” by the system in a 24-hours period, while theoretically possible, is not realistic. Therefore, the communications issues experienced by agencies could have been caused by or exacerbated by this artificiality. Nevertheless, the burden placed on the system simulated in this scenario remains plausible. In a major earthquake scenario, it is possible there would be more communication bandwidth used, and more operational areas receiving, coordinating, and sending mutual aid than occurred during this exercise.

Third, while we were able to recruit a large number of hospitals (n = 121), the overall hospital response rate was 26%, much lower than that of other recruited organizations. We found that small hospitals were particularly under-represented. If the response functions activated and communications challenges experienced vary by hospital size, then our characterization of how hospitals responded to the exercise scenario may be skewed. Several factors may have contributed to the low hospital response rate. First, because contact information for hospital preparedness coordinators is not publicly available, we conducted recruitment through a third party. Compared to governmental agencies, we had more limited control over hospital recruitment efforts—we were not able to directly contact potential survey respondents and to tailor recruitment efforts for non-responding hospitals. Second, respondents were asked to identify a primary hospital affiliation; however, we found that some respondents served as preparedness coordinators for entire hospital systems. As a result of our assigning one hospital per respondent, we are likely to have underreported the number of hospitals that are actually represented in our sample.

Lastly, these research results only provide us with a profile of capabilities and a characterization of communications challenges related to an IED scenario. We expect that, given a different scenario, the capabilities activated and communications challenges experienced might differ in important ways. As part of a larger research program at UC Berkeley, these variations will be documented and analyzed in a wide range of exercise and real event scenarios. Future research will focus not only on whether capabilities were activated, but also how central these activities were to the agencies’ objectives.

## Conclusions

By tapping into a pre-existing statewide operations-based exercise program in California, we accessed a large number of medical and health agencies with minimal cost. Using the 2010 Statewide Medical and Health Exercise in California as an opportunity to develop our research framework, we characterized several aspects of the public health and medical system’s response to a standardized emergency scenario. From a research perspective, this study provides a conceptual framework for improving the utility of exercise-based research. From a practitioner’s perspective, the results may provide a starting point for preparedness professionals’ dialogue about expected and actual organizational roles, responsibilities, and resource capacities within the public health system. Additionally, the identification of specific challenges to inter-organizational communications and information management offer specific areas for intervention.

## Endnotes

^a^ Operations-based exercises can be of two types: functional exercises and full-scale exercises. Functional exercises test inter-agency emergency operation centers’ command, control, and communications. In full-scale exercises, assets are deployed to the field.

^b^ An operational area consists of a County and its political subdivisions, such as cities and special districts.

^c^ An IED scenario is one of 15 National Planning Scenarios developed by the Department of Homeland Security in effort to establish a standard range of capabilities and resources necessary to respond to all potential high-impact events facing U.S. communities, states, and the nation.

^d^ Respondents who indicated their agency was a local EMS agency represent a mixture of the four legally-recognized types of agencies (i.e., local EMS agencies that: operate within a LHD, have joint powers authority, use county contractors, and operate within another department). However, we deferred to respondents’ agency classification.

^e^ Of 174 agencies, 23 were LHDs, 24 were local EMS agencies, 121 were hospitals, 4 were state agencies, and 2 indicated “Other”. Twelve of 24 local EMS agencies indicated their response represented both EMS and LHD for their jurisdiction; therefore, they were double-counted and contributed to the response rate for both agencies. The 5 RDMHC were not included in the overall study response rate because they were from local EMS agencies already accounted for.

^f^ Three acute psychiatric hospitals and three federal hospitals also responded to the survey. Although these responses are not included in the response rate calculation, they are included in the analysis of both research questions.

^g^ Of 144 agencies, 18 were LHDs, 21 were local EMS agencies, 103 were hospitals, 1 was a state agency, and 1 indicated “Other”. Twelve of 21 local EMS agencies indicated their response represents both EMS and LHD for their jurisdiction.

## Abbreviations

CHA: California Hospital Association; EMS: Emergency medical services; IED: Improvised explosive device; LHDs: Local health departments; RDMHC: Regional Disaster Medical and Health Coordinator/Specialist.

## Competing interests

The authors’ declare that they have no competing interests.

## Authors' contributions

JH, MP and TA conceived of the study and collaborated in the study design. JH and MP coordinated and implemented study recruitment and data collection, and coded qualitative data to analyze the questions about challenges to Communications and Information Sharing. JH, MP and TA developed the data collection tools. JY, JH, and TA participated in the quantitative data analysis and graphical display. All authors contributed to the interpretation of results, assisted in drafting the manuscript, and read and approved the final manuscript.

## Pre-publication history

The pre-publication history for this paper can be accessed here:

http://www.biomedcentral.com/1471-2458/12/680/prepub

## Supplementary Material

Additional file 1 **Supplementary Material. **Summary of the proportion of agencies activating key capabilities, by agency type. Click here for file

Additional file 2 **Figure S3. **Overall responses to the question, “During this exercise, what was your organization/agency’s most significant communication challenge?” were coded and classified into themes, categories and sub-categories. Multiple comments per respondent were possible. Figure S3 shows the number and percentage of overall comments (total number of statements = 176) that indicated a particular theme. An additional 15 respondents indicated “No communications challenges” and 8 responses were strictly related to the exercise design, as opposed to exercise play; these responses are not shown here. Click here for file

## References

[B1] Acosta J Nelson C Beckjord E Shelton S Murphy E Leuschner KJ Wasserman J A National Agenda for Public Health Systems Research on Emergency Preparedness RAND Corporation 2009 126 TR-660-DHHS

[B2] U.S. Department of Homeland Security Homeland Security Exercise and Evaluation Program (HSEEP) - Volume 1 2007 HSEEP Overview and Exercise Program Management, Washington, DC: Gov. Print. Off

[B3] Jackson BA Buehler JW Cole D Cookson S Dausey DJ Honess-Morreale L Lance S Molander RC O’Neal P Lurie N Bioterrorism with zoonotic disease: public health preparedness lessons from a multiagency exercise Biosecurity and Bioterrorism: Biodefense Strategy, Practice, and Science 2006 4 287 292 10.1089/bsp.2006.4.287 16999589

[B4] Dausey D Buehler J Lurie N Designing and conducting tabletop exercises to assess public health preparedness for manmade and naturally occurring biological threats BMC Public Health 2007 7 92 10.1186/1471-2458-7-92 17535426 PMC1894789

[B5] Biddinger PD Savoia E Massin-Short SB Preston J Stoto MA Public health emergency preparedness exercises: lessons learned Public Health Reports 2010 125 100 21133066 10.1177/00333549101250S514 PMC2966651

[B6] Nelson C Lurie N Wasserman J Assessing Public Health Emergency Preparedness: Concepts, Tools, and Challenges 2007 Public Health, Annual Reviews 10.1146/annurev.publhealth.28.021406.144054 17129174

[B7] Gebbie K Valas J Merrill J Morse S Role of exercises and drills in the evaluation of public health in an emergency response Prehospital and Disaster Medicine 2006 21 173 182 16892882 10.1017/s1049023x00003642

[B8] Lurie N Wasserman J Stoto M Myers S Namkung P Fielding J Valdez RB Local variation in public health preparedness 2004 Health Affairs, lessons from California hlthaff w4 10.1377/hlthaff.w4.341 15451958

[B9] California Statewide Medical and Health Training and Exercise Programhttp://www.californiamedicalhealthexercise.com/

[B10] California Department of Public Health (CDPH) California Emergency Medical Services Authority (EMSA) 2010 Statewide Medical and Health Functional Exercise Guidebook, Sacramento: CA

[B11] Mays G Public Health Services & Systems Research PHSSSR): Current State of the Field 2010 Northwest Center for Public Health Practice, University of Washington

[B12] U.S. Department of Homeland Security Target Capabilities List 2007 A companion to the National Preparedness Guidelines, Washington, DC: Gov. Print. Off

[B13] Yang JE Hunter JC Petrie M Aragon T Barriers and Facilitators to Agency Participation in the 2010 Statewide Medical and Health Exercise in California 2012 Cal PREPARE, Center for Infectious Diseases & Emergency Readiness, School of Public Health, UC Berkeley

