# Supplementary material for: Integrating a framework for conducting public health systems research into statewide operations-based exercises to improve emergency preparedness
Source: BMC Public Health. 2012 Aug 20;12:680. doi: 10.1186/1471-2458-12-680 (PMC3505730; doi:10.1186/1471-2458-12-680)
Supplement: Additional file 1 — Supplementary Material. Summary of the proportion of agencies activating key capabilities, by agency type. [file 1471-2458-12-680-S1.pdf]

Supplementary Material

The following section provides detailed results for both research questions described in the core paper.

Key Capabilities and Functions

Common Capabilities across Agency Types

As **Figure 2** and **Table 2** show, some capabilities were commonly activated by all types of medical and health agencies during the Statewide exercise. These common capabilities consist of *medical surge*, *medical supplies management and distribution*, *critical resource logistics and distribution*, *emergency operations center management*, and *intelligence gathering and recognition of indicators and warnings*. More than 30% of all agencies (excluding hospitals) engaged in *critical resource logistics and distribution*, and more than 40% of all agencies (excluding LHDs) were involved in *emergency operations center management*.

Emergency triage and pre-hospital treatment was commonly activated across local EMS agencies, local EMS agencies within a LHD, and hospitals, with 67%, 83% and 47%, respectively, reporting the capability. *Emergency public information and warning* was activated by 33% of LHDs and 42% of local EMS agencies within a LHD. *Responder safety and health*, *emergency public safety and security*, and *citizen evacuation and shelter-in-place* were also activated by at least one-third of local EMS agencies within a LHD and RDMHC.

Challenges to Inter-organizational Communications and Information Sharing

Most Significant Communications Challenge

As shown in **Figure 3**, 137 respondents (including 4 RDMHC) provided 176 comments when asked to describe the most significant communications challenge during the exercise. The majority (56%) of comments referred to challenges related to the complete or partial failure of communications equipment or systems—specifically an internet-based hospital status system which did not operate as expected, making it necessary for staff to collect data manually, repeatedly enter information, or rely on a back-up system (e.g., radio, fax, or runner) in order to receive or input their information. One respondent said,

"We were OK with our internal communications. However, when the [Internet-based hospital status] system became overloaded by the number of participating hospitals, the system experienced great problems and significant delays. It was frustrating for staff who tried several times to input the HAvBED [Hospital Available Beds for Emergencies and Disasters] information via the [Internet-based hospital status] system. HAM Radio communications were activated and the information was relayed."

Communication challenges were distributed similarly across the majority of all agency types, with the exception of RDMHC, who did not report any direct challenges with Communications Equipment/Systems. The majority (75%) of RDMHC reported their biggest communications challenge involved the inability to develop or maintain situational awareness. This capability was the second most significant communications challenge reported by hospitals (21%), LHDs (16%), and local EMS agencies (13%).

Inter-organizational Communications and Information Sharing

Respondents were also asked to identify all challenges to inter-organizational communications experienced by their organization, using a list of pre-identified challenges. These responses corroborate the results of the open-ended question described above. Commonly reported challenges to this question included: insufficient data systems capacity (38%) and untimely information (21%).

Figures

Figure 3 - Responses (overall) to “Most Significant Communications Challenge”

Overall responses to the question, “During this exercise, what was your organization/agency’s most significant communication challenge?” were coded and classified into themes, categories and sub-categories. Multiple comments per respondent were possible. Figure 3 shows the number and percentage of overall comments (total number of statements = 176) that indicated a particular theme. An additional 15 respondents indicated “No communications challenges” and 8 responses were strictly related to the exercise design, as opposed to exercise play; these responses are not shown here.

Tables

Table 2 - Ranking of Most Commonly Activated Key Capabilities, by Agency

Table 2 summarizes the percentage of agencies that activated various key capabilities (based on the Department of Homeland Security's Target Capabilities List) during the Statewide Exercise, and each key capability's ranking within each agency type. The proportion of each agency type that activated each key capability are sorted in descending order for LHDs, and all other agency types are organized accordingly. (Note: LHDs = local health departments, LEMSAs = local EMS agencies, and RDMHC = Regional Disaster Medical and Health Coordinators/Specialists).

	LHDs (n=18)	LEMSAs (n=9)	LEMSAs within a LHD (n=12)	Hospitals (n=103)	RDMHC (n=5)
**Key function**	**%**	**%**	**%**	**%**	**%**
Medical Surge	72	89	92	83	80
Medical Supplies Management and Distribution	56	67	92	53	60
Intelligence Gathering and Recognition of Indicators and Warnings	39	33	42	32	40
Critical Resource Logistics and Distribution	39	33	42	24	60
Emergency Public Information and Warning	33	22	42	17	20
Emergency Operations Center Management	28	67	58	43	60
Volunteer Management and Donations	28	22	58	15	40
Fatality Management	22	0	25	41	20
On-Site Incident Management	17	11	17	59	0
Responder Safety and Health	17	11	42	25	60
Environmental Health	17	0	50	8	40
Isolation and Quarantine	17	0	17	7	20
Emergency Triage and Pre-Hospital Treatment	17	67	83	48	20
Intelligence Analysis and Production	11	0	33	10	0
Epidemiologic Surveillance and Investigation	11	0	58	1	20
Emergency Public Safety and Security	11	22	33	23	60
CBRNE Detection (Chemical, Biological, Radiological, Nuclear, Explosive)	6	0	33	7	0
Mental health/Psychological Support	6	0	25	22	80
Explosive Device Response Operations	6	0	8	31	20
WMD and Hazardous Materials Response and Decontamination	6	0	25	8	0
Citizen Evacuation and Shelter-in-Place	6	0	33	13	60
Mass Prophylaxis	6	11	17	2	0
Mass Care (Sheltering, Feeding and Related Services)	6	11	8	20	0
Economic and Community Recovery	6	0	0	5	0
Other	6	22	0	1	40
Counter-Terror Investigation and Law Enforcement	0	0	0	9	0
Critical Infrastructure Protection	0	11	8	29	0
Food and Agriculture Safety and Defense	0	0	17	5	20
Laboratory Testing	0	0	8	6	0
Animal Disease Emergency Support	0	0	8	0	20
Fire Incident Response Support	0	0	8	6	0
Search and Rescue (Land-Based)	0	0	0	4	0
Structural Damage Assessment	0	0	0	14	0
Restoration of Lifelines	0	0	0	2	20
