# Supplementary figures and images for: Integrating a framework for conducting public health systems research into statewide operations-based exercises to improve emergency preparedness
Source: BMC Public Health. 2012 Aug 20;12:680. doi: 10.1186/1471-2458-12-680 (PMC3505730; doi:10.1186/1471-2458-12-680)

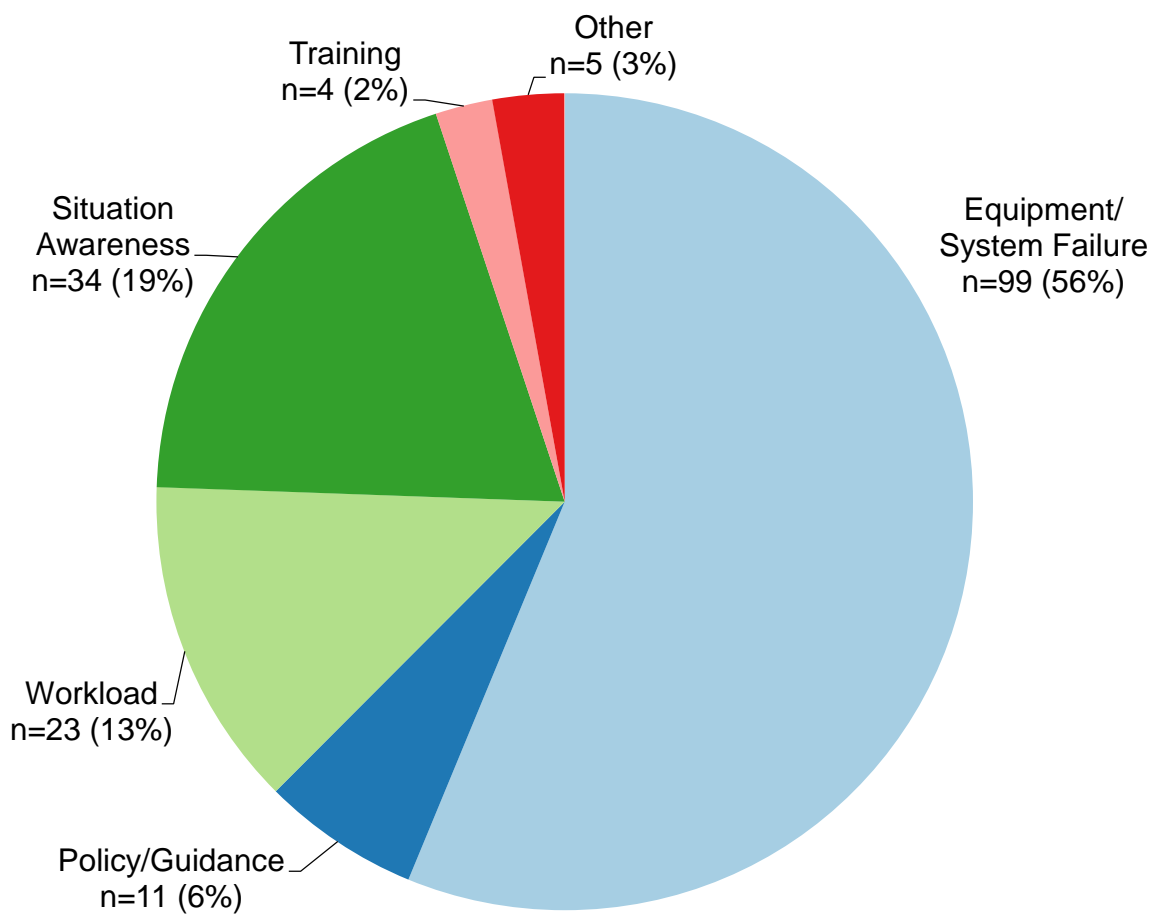

Supplement: Additional file 2 — Figure S3. Overall responses to the question, “During this exercise, what was your organization/agency’s most significant communication challenge?” were coded and classified into themes, categories and sub-categories. Multiple comments per respondent were possible. Figure S3 shows the number and percentage of overall comments (total number of statements = 176) that indicated a particular theme. An additional 15 respondents indicated “No communications challenges” and 8 responses were strictly related to the exercise design, as opposed to exercise play; these responses are not shown here. [file 1471-2458-12-680-S2.pdf]
